# Supplementary material for: Predicting factors that determine patients’ satisfaction with post-operative pain management following abdominal surgeries at Komfo Anokye Teaching Hospital, Kumasi, Ghana
Source: PLoS One. 2021 May 25;16(5):e0251979. doi: 10.1371/journal.pone.0251979 (PMC8148314; doi:10.1371/journal.pone.0251979)
Supplement: Attachment — Submitted filename: Response to Reviewers.docx [file pone.0251979.s002.docx]

**Department of Nursing**

**College of Health Sciences**

**Kwame Nkrumah University of Science and Technology**

**Kumasi-Ghana**

**29^TH^ March, 2021**

**Editor-in-Chief**

**PLOS ONE**

Dear Sir/Madam,

**RESPONSE TO REVIEWERS’ AND EDITORIAL COMMENTS: PONE-D-20-32363**

We express our appreciation to the editors and reviewers for the comments and suggestions made in reference to the manuscript that we submitted titled: *“Predicting factors that determine patients’ satisfaction with post-operative pain management following abdominal surgeries at Komfo Anokye Teaching Hospital, Kumasi, Ghana”*. Authors have revised the manuscript using the reviewers’ and the editorial comments.

Accordingly, we have provided the response to the comment in the table of changes below, and where the addition has been effected.

Thank you

**PRISCILLA FELICIA TANO**

**CORRESPONDING AUTHOR**

| **Reviewer #** | **Page Number** | **Reviewers’ Comments** | **Authors’ Responses** |
| --- | --- | --- | --- |
| **REVIEWER 1** |  | The first paragraph of materials and methods is mainly disconnected and there is no integrity of meaning. To understand the method of this study, it is necessary to read the manuscript three to four times. Materials and methods section should be revised.    The current results of the study were not mentioned in the discussion section.  The literature was well searched, but no correlation was made with the results found in the study or the differences of the study from the literature were not mentioned in the section. | We thank the reviewer for this important comment to revise the Materials and methods section. This comment has been addressed. Materials and methods section have been revised from page 6-9, line 125-197  We are grateful to the reviewer for this significant comment to mention the current result of the study in the discussion section. This comment has been addressed in the discussion section in page 18-22, line 284-363.  We appreciate this important comment to correlate or bring out the difference with the result found in the study and the literature. This comment has been addressed in the discussion section in page 18-22, line 294-295, 301-302, 324-316, 339-340, 345-346, 356-357. |
| **Reviewer 2;** |  | The period when the patients were operated on is not mentioned in the article.  The period when patients are operated on should be specified in Abstract (Methodology) and Main Text (Materials and Methods) sections | We thank the reviewer for this valuable feedback. The authors have included the periods when patients were operated on in the Abstract (Methodology) in page 3 line 58 and also in Materials and methods section in page 6, line 137. |
